# Association between Heavy Metal Exposure and Parkinson’s Disease: A Review of the Mechanisms Related to Oxidative Stress

**DOI:** 10.3390/antiox11122467

**Published:** 2022-12-15

**Authors:** Sarita Pyatha, Haesoo Kim, Daeun Lee, Kisok Kim

**Affiliations:** College of Pharmacy, Keimyung University, Daegu 42601, Republic of Korea

**Keywords:** heavy metals, oxidative stress, iron, mercury, copper, manganese, lead, Parkinson’s disease

## Abstract

Parkinson’s disease (PD) is a gradually progressing neurodegenerative condition that is marked by a loss of motor coordination along with non-motor features. Although the precise cause of PD has not been determined, the disease condition is mostly associated with the exposure to environmental toxins, such as metals, and their abnormal accumulation in the brain. Heavy metals, such as iron (Fe), mercury (Hg), manganese (Mn), copper (Cu), and lead (Pb), have been linked to PD and contribute to its progression. In addition, the interactions among the components of a metal mixture may result in synergistic toxicity. Numerous epidemiological studies have demonstrated a connection between PD and either single or mixed exposure to these heavy metals, which increase the prevalence of PD. Chronic exposure to heavy metals is related to the activation of proinflammatory cytokines resulting in neuronal loss through neuroinflammation. Similarly, metals disrupt redox homeostasis while inducing free radical production and decreasing antioxidant levels in the substantia nigra. Furthermore, these metals alter molecular processes and result in oxidative stress, DNA damage, mitochondrial dysfunction, and apoptosis, which can potentially trigger dopaminergic neurodegenerative disorders. This review focuses on the roles of Hg, Pb, Mn, Cu, and Fe in the development and progression of PD. Moreover, it explores the plausible roles of heavy metals in neurodegenerative mechanisms that facilitate the development of PD. A better understanding of the mechanisms underlying metal toxicities will enable the establishment of novel therapeutic approaches to prevent or cure PD.

## 1. Introduction

Parkinson’s disease (PD) is the most common neurodegenerative movement disorder, initially characterized by a decline in the coordination of movement. Additionally, PD is characterized by non-motor characteristics such as autonomic dysfunction and neuropsychiatric symptoms. It is the second most common neurodegenerative disorder after Alzheimer’s disease (AD). The worldwide prevalence of PD is likely to double by 2040 [1], which will make it by far the fastest-growing neurodegenerative disease, exceeding the increase in incidence of AD [2]. PD belongs to a group of neurological disorders characterized by common pathological hallmarks such as the disturbance of the dopamine circuit in the substantia nigra (SN) [3] and the presence of Lewy bodies in the dopaminergic neurons. The selective degeneration of dopaminergic neurons in the SN causes dopamine deficiency. Different mechanisms, including oxidative stress, excitotoxicity, mitochondrial malfunction, autophagy, misfolding, and the aggregation of specific proteins, are responsible for the alteration or death of nigrostriatal dopaminergic neurons in PD [4].

Although the etiological factors of PD have not been clearly elucidated, some cases of PD have been reported to be associated with metal poisoning [5], cerebrospinal meningitis [6], and genetic factors. Most (>90%) PD cases are related to environmental exposures, with heritable factors accounting for only 5–10% of PD cases [7]. These findings indicate that environmental factors play a significant role in the etiology of PD. A study conducted on a population of 19,842 twins concluded that PD is affected by environmental factors, and that genetic factors do not play a major role in the development of the disease [8]. Exposures to pesticides [9], polymers [10], and other environmental toxicants, such as metals and solvents [11], are related to an increased risk for PD. Heavy metals, such as iron (Fe), manganese (Mn), mercury (Hg), lead (Pb), and copper (Cu), have been identified as potential causes of PD as well as likely contributors to the disease progression [11]. The epidemiological studies that associate metal exposure with an increased probability of PD onset are shown in Table 1.

**Table 1:** The epidemiological studies that associate metal exposure with an increased probability of PD.

Metal	Type of Study	Sample Size (Case/Control)	Geographical Region	Main Findings	Reference
Fe, Cu, Pb	Case-control	150/170	India	Level of metals in plasma was positively correlated with PD: Fe (r = 0.29, *p* < 0.001), Pb (r = 0.16, *p* = 0.007), and Cu (r = 0.11, *p* = 0.047)	[12]
Fe, Mn, Cu, Pb	Case-control	144/464	USA	Exposure exceeding 20 years of exposure to Mn (OR = 10.61, 95% CI = 1.06, 105.83) and Cu (OR = 2.49, 95% CI = 1.06, 5.89) was associated with PD. Combination of Pb and Cu (OR = 5.24, 95% CI = 0.59, 17.21), Pb and Fe (OR = 2.83, 95% CI = 1.07, 7.50), and Fe and Cu (OR = 5.24, 95% CI = 1.40, 9.71) was associated with PD	[13]
Pb	Case-control	121/414	USA	Pb exposure for lifetime exposure increase for PD (OR = 2.27, 95% CI = 1.13, 4.55).	[14]
Pb	Case-control	330/308	USA	Compared with the lowest quartile of tibia Pb, the OR for PD in the highest quartile was 3.21 (95% CI = 1.17, 8.83)	[15]
Hg	Case-control	54/95	Singapore	The logarithmic unit elevate in blood and urine Hg is associated with 21.0 (*p* < 0.05) or 18.65 times increase in risk of PD.	[16]
Hg	Case-control	17/15	Taiwan	A significantly negative correlation between urine Hg level and uptake ratio in the striatum, caudate, putamen (r = −0.501, *p* = 0.040; r = −0.635, *p* = 0.006; r = −0.559, *p* = 0.020, respectively)	[17]

PD, Parkinson’s disease; OR, odds ratio; CI, confidence interval.

Chronic exposure to heavy metals is linked with the activation of proinflammatory cytokines, resulting in neuronal loss through neuroinflammation. Metals disrupt redox homeostasis while inducing free radical generation and decreasing antioxidant levels [18]. In addition, the huge increase in oxidative stress associated with such exposure compromises the activity of the ubiquitin proteasome system, resulting in protein aggregation. These protein aggregates disrupt cellular processes, resulting in cell death [19]. Metal concentrations in the brain of deceased PD patients have been found to be significantly altered compared to age-matched non-PD controls, suggesting that exposure to metals is strongly associated with the incidence of PD [20]. Moreover, the accumulation of certain metals, including Mn, Hg, or even excessive Fe consumption, may play a role in the development of non-depressive PD [21]. High Fe concentration induces oxidative damage in PD, and metal-induced oxidative damage has been implicated as a cause of PD [12]. Despite the importance of metals in the development of PD, there have been few review articles comparing the oxidative stress of major metals on the pathogenesis of PD. Here, we review the relations between single and mixed exposure to major metals and the prevalence of PD.

## 2. Metals and Parkinson’s Disease

The roles of metals in the pathogenesis and progression of PD have been studied extensively. Exposure to toxic metals and the depletion of essential metals in the body contribute significantly to the etiology and pathological processes of PD. For example, Fe deficiency is related to the malfunction of the peripheral or central neurological system and decreases in dopamine and serotonin levels in the brain, giving rise to PD with restless leg syndrome [22]. In addition, the accumulation of Fe in the gray matter nuclei of the basal ganglia and midbrain has been suggested to play a role in the etiology of PD [23]. Many epidemiological studies have demonstrated an association between PD and exposure to metals, such as Hg, Cu, Mn, Pb, aluminum (Al), bismuth (Bi), and zinc (Zn) [13,24]. One study showed that the risk for PD is increased by 2–10 times among workers with occupational exposure to Mn, Pb, or Cu for periods exceeding 20 years [11].

### 2.1. Iron and Parkinson’s Disease

**Figure 1:**
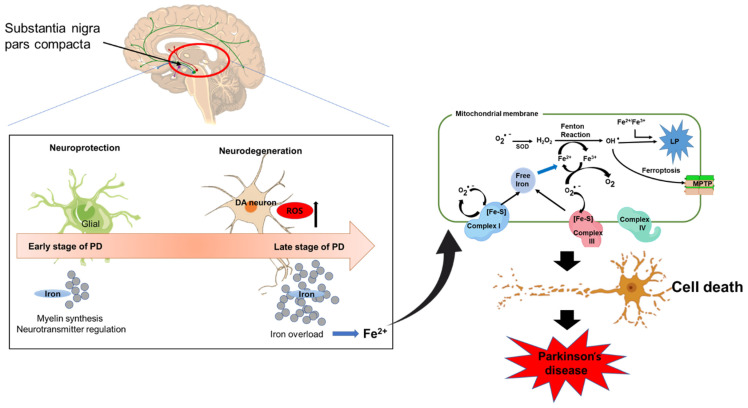
Role of Fe in mitochondrial dysfunction and neuronal death leading to PD. Increased levels of Fe may cause neurodegeneration through the production of large amounts of reactive oxygen species (ROS) via the Fenton reaction. Fe can induce mitochondrial oxidative stress through interactions with different ROS. Free Fe can be released from mitochondrial Fe-sulfur clusters in complexes I and III upon interacting with ROS. The redox pair Fe^2+^-Fe^3+^ can directly stimulate lipid peroxidation (LP), which is an indicator of oxidative stress and contributes to mitochondrial dysfunction via mitochondrial permeability transition pore (mPTP) formation, thus leading to neural damage and the induction of PD.

Fe is one of the most common metals with a role as a necessary cofactor in various biological functions, including DNA synthesis, mitochondrial respiration, oxygen transportation, and synthesis of neurotransmitters. Fe concentrations are higher in the SN and globus pallidus of the healthy brain [25], while the midbrain, cerebellum, cortical gray matter, and white matter have lower Fe concentrations [26]. The regions of the brain that are involved in motor functions appear to have higher concentrations of Fe than those regions that are not involved in motor functions. This explains the relationship between Fe loading and motor-associated disease [27]. In addition, cognitive dysfunction and the reduction in myelin formation by neurons are associated with changes in the level of Fe in specific parts of the brain [28]. Fe accumulation is especially high in the SN in the PD brain [29,30]. The high accumulation of Fe in the SN is neurotoxic [31]; at same time, Fe also plays a crucial part in dopamine synthesis, as it functions as a cofactor for tyrosine hydroxylase, an enzyme that limits the production of chemical transmitters [32]. So, an excess availability or insufficiency in the level of Fe can be harmful to dopaminergic neurons. Furthermore, in the progression of PD, both glial cells and dopamine neurons are involved in iron metabolism. Myelin synthesis and neurotransmitter regulation may provide neuroprotection during early stages of PD. However, increased levels of Fe may cause neurodegeneration through high levels of ROS (Fenton reaction) during the late stage of PD [33]. Therefore, an imbalance in the levels of metals, especially Fe, can be hazardous for dopaminergic neurons (Figure 1).

Neuromelanin, known for its antioxidant properties, protects against Fe-induced dopamine toxicity when Fe concentrations are low, but acts as a pro-oxidant when Fe concentrations are high [34]. Neuromelanin has also been proposed as an Fe storage molecule. A significant Fe level has been detected as a neuromelanin-bound form in SN neurons using the X-ray fluorescence technique [35]. The neuromelanin from the human SN has both high- and low-affinity ferric (Fe^3+^) binding sites, and neuromelanin-bound Fe is redox-active [36]. The disintegration of the neuromelanin–Fe complex may be linked with oxidative stress in PD [37].

Fe-dependent cell death pathways, known as ferroptosis, have been discovered in dopaminergic neuron models and in 1-methyl-4-phenyl-1,2,3,6-tetrahydropyridine (MPTP) mice, a well-known animal model of PD [38]. Ferroptosis is a type of cell death defined by Fe- and reactive oxygen species (ROS)-dependent events that result in lipid peroxidation and cell membrane rupture [39]. Ferroptosis involves several molecular processes that are associated with PD. These events, such as Fe overload, mutations in Fe transport proteins, mitochondrial malfunction, decrease in glutathione (GSH) level, and the build-up of lipid peroxidation products, have been identified in ferroptosis-mediated cell death, and are also seen in PD [40]. Furthermore, excess Fe in the SN and globus pallidus results in dopaminergic neuronal death by generating hydroxyl radicals (·OH) through the Haber–Weiss and Fenton reactions, which compromise the structural stability of neurons [41]. α-Synuclein, an extremely acidic protein, facilitates the binding of Fe as ferritin with neurofilaments. Recently, Abeyawardhane et al. reported evidence to support the ferrireductase activity of α-synuclein upon binding to Fe^3+^ under anaerobic conditions as well as an increase in the antiparallel β-sheet composition, as is characteristic of α-synuclein aggregates formed under oxidizing conditions in the presence of ferritin [42]. It also interacts with intracellular Fe(II) to form aggregates of α-synuclein [43].

### 2.2. Manganese and Parkinson’s Disease

**Figure 2:**
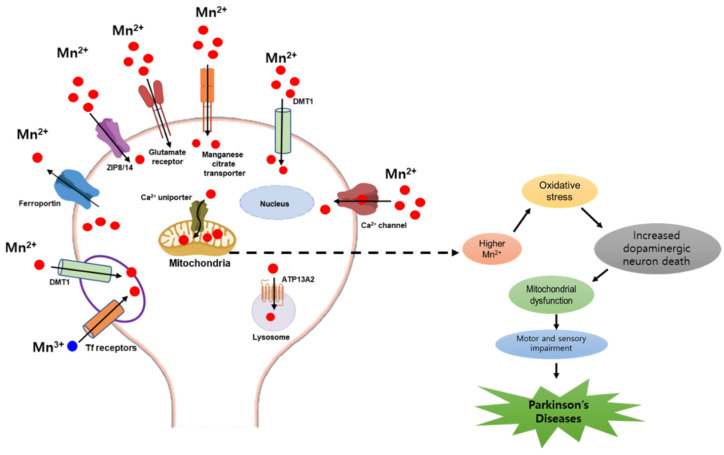
Receptors and channels involved in Mn homeostasis. Various cellular receptors, such as divalent metal transporter 1 (DMT1) and transferrin receptor (TfR), as well as Ca^2+^ channels, ZIP8/14 transporter, and Mn citrate transporter facilitate the entry of divalent Mn into cells, whereas ferroportin and Ca^2+^ facilitate its expulsion from cells and mitochondria, respectively. Mn^2+^ is passively transported via glutamate-activated ion channels, while Mn^3+^ entry is facilitated by transferrin. All of these mechanisms enhance the trafficking of Mn^2+^ across the cells, leading to mitochondrial dysfunction and increased dopaminergic neuronal death, resulting in PD.

Mn is an essential micronutrient for healthy growth and development, with roles in immune defense, bone formation, the maintenance of cellular energy balance, and the prevention of free radical formation [44]. Multiple enzymes, such as glutamine synthetase (GS), Mn superoxide dismutase (SOD), arginase, serine/threonine protein phosphatase I, and pyruvate decarboxylase, use Mn as a cofactor to regulate various biochemical and cellular events that are necessary for the production of neurotransmitters and neuronal and glial cell function [45]. Mn is the fourth most extensively utilized heavy metal, which has 11 oxidation states [46]; the most common oxidation states of Mn are Mn^2+^ and Mn^3+^ in biosystems [47], and these two also are the most common forms present in the human body. As Mn^2+^ is more chemically stable in the body than Mn^3+^, Mn is mostly absorbed into metalloenzymes in the Mn^2+^ form [48]. Mn travels to the central nervous system (CNS) through cerebrospinal fluid (CSF) or the bridging cerebral capillary endothelial membrane [49]. Several mechanisms, including enhanced diffusion and active transport by the divalent metal transporter 1 (DMT1), ZIP8, and the transferrin receptor system, play important roles in crossing the blood–brain barrier (BBB) [50]. Generally, Mn^3+^ is transported to the central nervous system (CNS) through cerebrospinal fluid (CSF) or the bridging cerebral capillary endothelial membrane. Transferrin (Tf) is the plasma transporter protein for Mn^3+^, and divalent metal transporter (DMT) or Tf receptor-mediated endocytosis are two mechanisms by which Mn^3+^ enters neurons [51,52]. In the case of Mn^2+^ toxicity, the accumulation of Mn in dopamine (DA)-rich areas is facilitated by DMT1 [53]. According to several studies, Mn^2+^ can transport to the BBB through the voltage-gated and Ca^2+^ channels [54], ionotropic glutamate receptor Ca^2+^ channels [55], and a Mn citrate transporter [56]. Additional evidence has reported that Mn^2+^ is transported by the Zn transporters ZIP8 and ZIP14 [57] as well as ATP13A2, a P-type transmembrane ATPase protein [58]. Furthermore, ferroportin and Ca^2+^ facilitate its expulsion from cells and mitochondria, respectively [59]. Moreover, the presence of the mitochondrial Ca^2+^ uniporter is responsible for fast and electrogenic Ca^2+^ and Mn^2+^ uptake to mitochondria [51]. Mn^2+^ is very difficult to transport out of brain mitochondria after being sequestered within the mitochondria by rapid uniporter mechanisms. In addition, this also explains why Mn has a relatively long half-life in the brain [51]. All of these mechanisms enhance the trafficking of Mn^2+^ across the cells, leading to mitochondrial dysfunction and increased dopaminergic neuronal death, resulting in PD. Despite its essential physiological roles, Mn also has neurotoxic effects, mostly at high concentrations and with prolonged exposure. Indeed, Mn has been shown to hamper dopaminergic, glutamatergic, and GABA transmission, and to induce oxidative stress, mitochondrial dysfunction, and neuroinflammation (Figure 2).

The accumulation of Mn in the basal ganglia activates glutamate receptors to increase postsynaptic glutamate receptor sensitivity [60]. Through this process, it is thought to generate aberrant pallidal neuron activation, which leads to motor dysfunction. In addition, the accumulation of Mn in the brain reduces the rate of removal of glutamate by astrocytes in the synapses and increases the sensitivity of glutamate receptors, resulting in mitochondrial malfunction, oxidative damage, and subsequent neuronal loss [45]. Intracellular Mn accumulates mostly as Mn^2+^ in the mitochondria, through the calcium (Ca^2+^) uniporter. Increased Mn levels in mitochondria constrain oxidative respiration, resulting in ROS production and eventually the induction of mitochondrial malfunction [61]. The potential of Mn to boost oxidative stress is due to its oxidative state change from Mn^2+^ to Mn^3+^, further enhancing its pro-oxidant characteristics [62]. Mn hinders mitochondrial electron transport, increases free radical production, and causes α-synuclein protein aggregation [51]. The accumulation of Mn in neurons occurs mainly in the globus pallidus of the basal ganglia, SN, and subthalamic nucleus, followed by the striatum, resulting in progressive neuronal degeneration [63,64]. The disturbance of basal ganglia function is also seen in PD. Mn toxicity is characterized by motor and sensory disturbance, where motor impairment is seen as generalized bradykinesia, tremor, cock-walk gait, and widespread rigidity, similar to PD [65]. Several studies have investigated the possible association between chronic Mn overexposure and PD, showing that there is a direct relation between occupational exposure to Mn and PD, particularly in workers exposed to Mn for periods longer than 30 years [66]. A longitudinal cohort of 886 American welding workers showed that cumulative Mn exposure accelerated the course of parkinsonism [67]. A geographical study also showed that higher occurrence rates of Mn-induced parkinsonism features and accompanying mortality are related to areas in the USA with significant industrial Mn emissions.

A number of studies have reported a link between high Mn concentration in the brain and impaired dopaminergic neurotransmission. Though, a definite mechanism on how Mn leads to dopaminergic neurodegeneration has not been well postulated. One of the proposed ideas is Mn’s capacity to provoke ROS generation through quinone formation. Mn has been demonstrated to produce apoptosis in dopamine neurons in a caspase-3-dependent manner by stimulating protein kinase C delta [68]; this protein kinase C delta has been associated with neurodegenerative diseases such as PD [69]. Moreover, there is evidence of an increase [70], decrease [71,72,73], or both [74] in tissue levels of dopamine following Mn exposure. Meanwhile, positron emission tomography (PET) and single-photon emission computed tomography (SPECT) examinations of PD patients’ dopaminergic neurons have revealed a gradual loss of dopamine transporter (DAT) and vesicular monoamine transporter 2 (VMAT2). Mn halts dopamine reuptake by mobilizing DAT receptors from the cell structure to intracellular compartments, prompting dopamine-generated cell toxicity [75]. In addition, a decrease in 3,4-dihydroxyphenylacetic acid activity has been detected by [18F]-fluoro-DOPA PET, along with normal or elevated levels of striatal dopamine receptor 2 [73,76,77]. Both Mn-induced parkinsonism and PD share similar symptoms, including widespread rigidity and generalized bradykinesia, along with common pathophysiological processes, including protein aggregation, impaired proteasomal function, oxidative stress, autophagy functions, excitotoxicity, mitochondrial dysfunction, abnormal signal transduction, and cell death pathways.

### 2.3. Mercury and Parkinson’s Disease

Hg is a transition metal created by natural phenomena, such as volcanic eruptions and evaporation, as well as anthropogenic sources, such as pollution from coal-fired power plants and incinerators. Hg exists in various chemical forms, including elemental mercury (Hg^0^), organic mercury (MeHg), and inorganic mercury (Hg^2+^ and Hg^+^). Although the effects of human exposure to all forms of Hg have been investigated, MeHg exposure is of particular interest in the context of PD. Inorganic Hg is the most toxic form of Hg found in high concentrations in aquatic environments [78] and it also shows an elevated rate of penetration into the CNS, making it a significant neurotoxicant [79]. Due to its efficient delivery into the brain, the CNS is a major target organ of Hg poisoning. Hg passes across the BBB and its uptake by brain cells occurs through the MeHg–L-cysteine complex via the neutral L-type amino acid carrier [80]. It disrupts protein synthesis and biological components in the growing brain by impairing ribosomal activity and the function of the endoplasmic reticulum [81]. The detection of high levels of Hg in the brains of fetal mice exposed directly to MeHg during pregnancy suggested the efficient transplacental transfer of MeHg and also high transport into the developing brain [82].

Hg is also involved in many neural processes, including oxidative stress, reducing GSH levels, mitochondrial injury, and free radical aggregation [83]. Hg prevents metalloproteins from chemically combining with sulfur, which disables metalloproteins in the intestinal cell membrane that would otherwise bind cuprous ions, and as a result, free Cu induces toxicity, and the formation of Zn-Cu–SOD complexes is disturbed. Furthermore, it induces the displacement of Zn in SOD and metallothionein (MT), resulting in neurotoxicity [84]. Hg has also been reported to increase TNF-α concentration, which stimulates cellular apoptosis and neuroinflammation leading to PD-like manifestations. Patients exposed to Hg (e.g., in dental amalgam fillings) are likely to have a six-fold increased risk for PD compared to nonexposed subjects [85].

**Figure 3:**
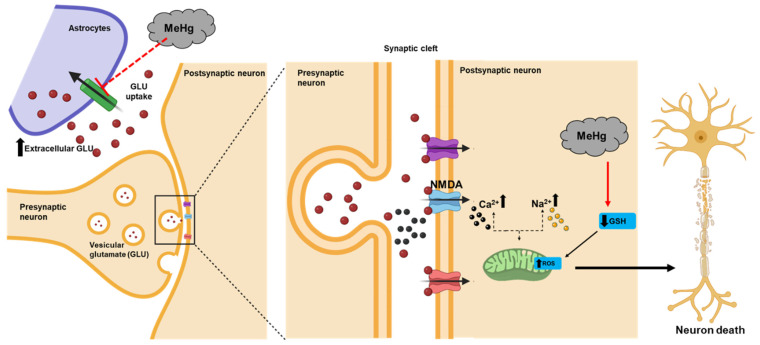
MeHg-induced glutamate and Ca^2+^ dyshomeostasis and oxidative stress involving neuronal death. MeHg inhibits astrocytic glutamate uptake and enhances glutamate release from presynaptic terminals. The increased extracellular glutamate levels lead to the overactivation of N-methyl-D-aspartate (NMDA)-type glutamate receptors, enhancing the influx of Ca^2+^ into postsynaptic neurons. The increased levels of intracellular Ca^2+^ may cause mitochondrial dysfunction and increased reactive oxygen species (ROS) formation. MeHg disrupts the redox state of the cells, which indirectly depletes glutathione (GSH), which will further increase ROS production in both astrocytes and neurons.

Hg is a heavy metal that causes brain toxicity and plays a role in the development of neurodegenerative diseases, such as PD. There are several similarities between the outcome of Hg exposure and the manifestations of PD, including GSH depletion in the SN, loss of dopamine receptors, glutamate elevation, and mitochondrial dysfunction [8] (Figure 3).

Several in vitro [86] and in vivo studies [87,88] have reported that exposure to MeHg results in the depletion of GSH, which plays an important role in the preservation of redox homeostasis [89]. Various aspects of neurotoxicity induced by exposure to MeHg have been described in relation to decreased GSH [90]. Decreased GSH levels have also been reported in the cerebrum and cerebellum of animals exposed to low doses of MeHg in the micromolar range [91], indicating that not only MeHg–GSH interactions but also the generation of ROS through GSH-dependent mechanisms are responsible for MeHg-induced GSH oxidation [92]. In addition, the generation of ROS induces protein oxidation, where MeHg-mediated ROS modulates the redox states of proteins with consequent effects on their activities. The function of astrocytic glutamate transporters is downregulated by hydrogen peroxide produced by MeHg [93]. Glutamate is a crucial excitatory neurotransmitter, which has direct and indirect pro-oxidative effects. MeHg increases the extracellular glutamate concentration by inhibiting glutamate uptake [94] and stimulating glutamate delivery into the synaptic cleft [95] (Figure 3). In studies that used cultured astrocytes, exposure to Hg^2+^ resulted in decreased glutamate uptake and increased glutamate release as well as the intracellular influx of Na^+^ and Ca^+^ due to the overstimulation of the N-methyl-D-aspartate (NMDA) receptor, which is a subtype of glutamate receptor involved in ROS production and neurotoxicity [96,97]. The elevated intracellular Ca^+^ levels stimulate neuronal nitric oxide synthesis, resulting in the overproduction of nitric oxide and mitochondrial death [90].

A study reporting the significance of the DJ-1 protein in Hg ion binding showed that a mutated version of the PARK7 gene encoded a DJ-1 protein that had lost its ability to bind metal ions, resulting in higher Hg toxicity [24]. In a study that investigated the relationship between PD and Hg levels, Hg levels in the blood of PD patients were six-fold higher than in healthy controls, suggesting that Hg is an important factor in the etiology of PD [98]. In addition, a study of the association between occupational Hg exposure and PD presented a case in which a patient with occupational exposure to Hg vapor for 33 years developed typical symptoms of PD, including bradykinesia, spatial disorientation, memory loss, tremor, paresthesia of the upper extremities, and cortical and cerebellar atrophy [99].

### 2.4. Copper and Parkinson’s Disease

**Figure 4:**
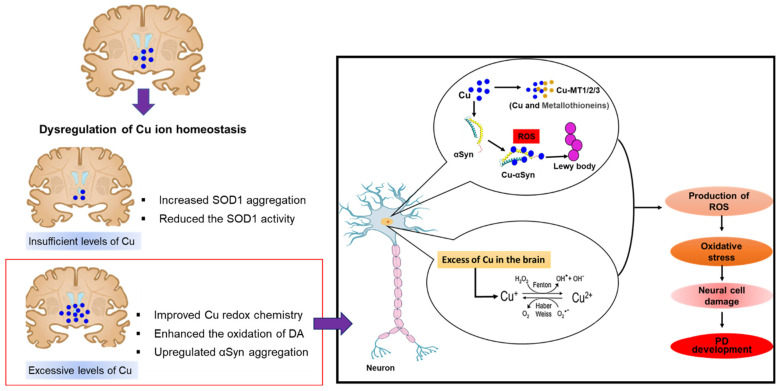
Dysregulation of Cu ion homeostasis in PD. Excessive levels of Cu improved dopamine (DA) oxidation, upregulated α-synuclein (αSyn) aggregation, and formation of Lewy bodies. Excess Cu levels in the brain take part in Fenton chemistry as a catalyst to produce free radicals. Both processes lead to ROS production causing dopaminergic neuronal death and PD.

Cu is the third most prevalent essential metal in the human body and is present in the highest levels in the liver and brain [100,101]. Cu is a trace element that is vital for enzyme function in the generation of mitochondrial energy and neurotransmitter processing, and it also participates in various physiological functions, including the maintenance of the stability of blood vessels and myelination, Fe homeostasis, and antioxidant protection [102,103]. The involvement of Cu as a cofactor or structural component in various cuproproteins makes it crucial to a range of biological activities. The oxidative state of Cu can be from Cu (I) to Cu(II), and this characteristic is precisely synchronized by the protein structure that binds the metal ion [104]. Cu also plays a role in Fe homeostasis through interactions with ceruloplasmin, which shows Cu-based oxidase activity. In addition, Cu plays significant roles in synapses through its interactions with neurotransmitter receptors and synaptic proteins. Cu is involved in various neuronal cell activities by influencing synaptic activity, signaling cascades, and excitotoxic cell death triggered by neurotrophic substances [105]. Given the importance of Cu, especially in cellular pathways, alterations in the homeostatic system resulting in either increases or decreases in intracellular Cu levels can cause serious neurological disorders related to PD. In addition, cellular pathways linked to either excess or deficit of Cu concentration result in increased oxidative stress, suggesting that a stressful intracellular environment can induce cell damage in both conditions [105]. Furthermore, Cu induces dopamine oxidation and α-synuclein aggregation, leading to dopaminergic neuronal death and PD (Figure 4).

Cu is a transition metal that takes part in Fenton chemistry as a catalyst to produce free radicals [106]; it is also involved in the production of hydrogen peroxide. As hydrogen peroxide is produced as a consequence of monoamine oxidase metabolism, this is especially significant in areas of the brain that process biological amines such as dopamine. Several studies [107,108,109] have reported increased Cu levels in the CSF of PD patients compared to controls. They also suggested that free Cu in the CSF may be linked to clinical factors, and it is a potential biochemical marker of PD [108].

Although the precise pathophysiology of PD is still unclear, there is widespread agreement that oxidative stress and mitochondrial dysfunction play roles in the disease progression. Postmortem investigations of the PD brain have revealed high levels of oxidative stress on proteins, lipids, and DNA, and mitochondrial malfunction related to the disruption of complexes I and III [110]. Furthermore, neurodegeneration of the SN due to oxidative damage and mitochondrial malfunction has also been reported. Free Cu can also be toxic due to its linkage with cysteine residues in protein, which can inhibit enzymatic activity [111]. Dopamine receptor 2 binding sites have been shown to be decreased in rats treated with metal cations (Cu^2+^, Hg^2+^, and Cd^2+^), with increased reactivity regarding thiols. In one study, a significant decrease of 40–60% was observed following the administration of 3 mM Cu, suggesting that Cu-induced thiol changes may have functional effects [112]. Cu also causes oxidative damage by participating in a noxious redox chemical process that engages oxidative derivatives. As shown in Figure 4, Cu may also be involved in the Fenton and Haber–Weiss reactions, which convert superoxide anions and hydrogen peroxide to hydroxyl radicals [110]. Cu promotes dopamine oxidation, resulting in the production of a range of potentially hazardous species, such as dopamine quinones, hydroxyl radicals, O_2_^−^, and H_2_O_2_. The concentration of Cu in the brain is highest in the SN, where the majority of neuromelanin is present [113]. The presence of Cu in neuromelanin shows that it is involved in the oxidative polymerization of dopamine [114]. In addition, oxidation can also be initiated by the attachment of Cu ions to ligands or proteins associated with neurodegeneration [114].

In vitro studies have demonstrated that millimolar concentrations of different metal ions, including Cu, promote the development of the partially folded amyloidogenic conformation of amyloid-β protein that is more likely to undergo aggregation [115]. Even at the physiologically appropriate concentration, Cu has been shown to accelerate the development of α-synuclein fibrils without changing their morphology [116].

Not only does increasing the concentration of Cu affect disease incidence, but a decrease in Cu concentration is also associated with an increased likelihood of disease occurrence or progression. In PD patients, the Cu level and oxidase activity of blood ceruloplasmin and the number of Cu atoms per ceruloplasmin molecule are lower than in healthy subjects of the same age [117,118]. In addition, Cu concentrations in the SN and locus coeruleus of PD patients are reduced by 35–50% compared to healthy age-matched controls [119]. Cu boosts ferroxidase activity and contributes to Fe homeostasis by binding to ceruloplasmin, and may therefore contribute to indirect toxicity mediated by changes in Fe concentration [120]. The correlation between Cu exposure and the incidence of PD is supported by various epidemiological studies. Many epidemiological studies have reported that occupational exposure to Cu alone or in mixtures with other metals is associated with an increased risk of developing PD [121,122,123,124].

### 2.5. Lead and Parkinson’s Disease

Pb is a nonessential heavy metal that is recognized as one of the most harmful pollutants for human health [125]. Exposure to Pb has been increasing due to the wide variety of uses of lead, from industry to everyday life. Pb is toxic to various systems and organs in the body, including the nervous system, bones, kidneys, reproductive organs, intestines, and heart. Epidemiological studies have confirmed a two- to three-fold increased risk for PD associated with exposure to Pb, indicating a positive relationship between Pb exposure and PD [15]. A case–control study in the USA showed that individuals in the highest quartile of lifetime Pb exposure had double the risk for PD than those in the lowest quartile [14]. The mechanism by which Pb causes neurotoxicity is quite complex and involves many signaling processes. Children’s brains are more vulnerable to Pb exposure than those of adults, in part because of their immature BBB, which allows Pb to easily cross it [15]. Various observational and experimental studies have shown that Pb exposure at a young age may have long-term neurological, behavioral, and epigenetic effects, suggesting that Pb-exposed children are at significant risk of developing neurodegenerative diseases as they age.

**Figure 5:**
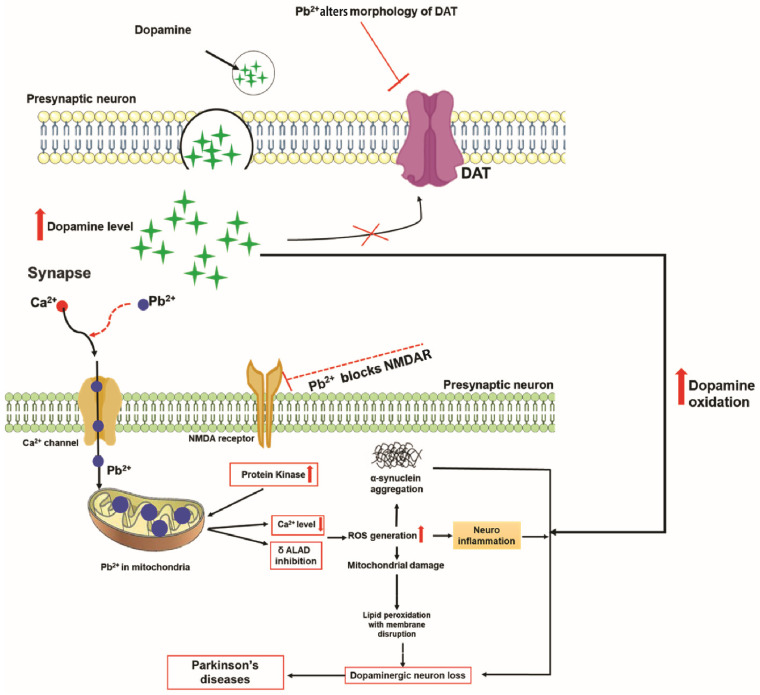
Pb^2+^ induces changes in dopamine transporter (DAT) morphology, leading to an increase in extracellular dopamine level, resulting in neurotoxicity via dopamine oxidation. Moreover, Pb^2+^ and Ca^2+^ share a permeability pathway represented by a Ca^2+^ channel and increased Pb^2+^ level in mitochondria, causing dopaminergic neuron loss. In addition, Pb^2+^ induces α-synuclein aggregation, NMDA receptor (NMDAR) blockade, and the activation of protein kinase C, leading to reduced Ca^2+^ release from mitochondria. Moreover, δ-aminolevulinic acid dehydratase (δ-ALAD) blockade by Pb^2+^ delivered by mitochondria leads to the initiation of lipid peroxidation. These mechanisms ultimately cause the depletion of dopaminergic neurotransmission resulting in the development of PD.

Pb is a toxic metal that can replace or mimic the action of calcium in the body, which is essential for neuronal signaling, myelination, and the functioning of glial cells [126] (Figure 5). When Pb crosses the BBB through calcium channels, it enters neurons and glial cells. Pb affects the release and reuptake of neurotransmitters, such as acetylcholine and dopamine, affecting many neurotransmission processes in the brain that depend on calcium ions [127]. In PD, Pb induces changes in DAT via damage to the morphology of dopaminergic neurons, and this may lead to an imbalance of extracellular dopamine levels, resulting in neurotoxicity in the CNS [128]. Moreover, Pb impairs the functional capacity of the cholinergic, dopaminergic, noradrenergic, and GABAergic systems. Dopaminergic neurotransmission is reduced by oxidative stress, mitochondrial dysfunction, and elevated gliofilaments in astrocytes after Pb exposure [129]. Pb affects energy metabolism via the blockade of NMDA receptor and the activation of protein kinase C, resulting in the inhibition of calcium delivery from mitochondria. As shown in Figure 5, this triggers the generation of ROS in the mitochondria, which leads to mitochondrial destruction and neuronal cell death [126].

In addition, Pb has recently attracted a great deal of attention as a major factor contributing to the pathogenesis of neurodegenerative diseases. Pb has been suggested to play a role in the modulation of gene regulation in Pb-exposed populations [130]. Early-life exposure to Pb disturbs gene expression and regulation, which are associated with the pathology of neurodegenerative diseases related to tau pathology [131]. Pb affects gene expression by reducing the activity of DNA methyltransferases in affected cells [132]. About 150 genes have been shown to be differentially expressed in mice exposed to Pb at a young age [133], and some of these genes are involved in pathological protein accumulation [134]. Pb has been shown to increase α-synuclein concentration and tau hyperphosphorylation in the hippocampus, resulting in the initiation of autophagy and apoptosis [135]. Pb toxicity is also exhibited via Fe regulatory channels in the presence of amyloid precursor proteins in SH-SY5Y neuroblastoma cells, an in vitro model of dopaminergic neurons. Protein kinase C has been reported to play a role in dopamine transport, and the activation of protein kinase C by Pb induces oxidative stress, thereby leading to neurotoxicity [136]. Furthermore, Pb-related toxicity is also caused by the blockade of delta-aminolevulinic acid dehydratase (δ-ALAD) as well as the accumulation of its substrate, δ-ALA, which quickly oxidizes to form free radicals, thus initiating the process of ferrous ion-induced lipid peroxidation [137] (Figure 5).

## 3. Metal Mixtures and Parkinson’s Disease

In many cases, humans are exposed to multiple or mixed metals rather than to a single metal. Metals are frequently introduced into the environment as mixtures, and mixtures of neurotoxic metals are widely present in the environment [138]. Occupational exposure, contaminated food and medical products, and environmental pollution are among the major sources of metal exposure. Therefore, there have been a number of epidemiological studies regarding the relationship between exposure to mixtures of metals, such as Pb, Mn, Cu, Hg, Al, Zn, Bi, and Fe, and the prevalence of PD. Mixed exposure to metals can occur even within the body via metal ions released from implants or dental restorations, which are phagocytosed by macrophages and transported to the brain [139]. Exposure to metals in combination with other environmental toxins, such as pesticides, herbicides, and fungicides, synergistically accelerates the aging-related loss of nigral dopaminergic neurons, aggregation of α-synuclein, and reduction of dopamine levels [140].

Mixed metal exposure can lead to competitive interactions with macromolecules due to their similarity in function, resulting in increased toxicity. In relation to PD, it has been reported that the effects of mixtures of Pb and Cu, Fe and Pb, or Cu and Fe are greater than those of the individual metals alone due to their synergistic effects [13]. There are significant similarities between the neurodegenerative changes exhibited by PD and neurotoxicity caused by Hg exposure. The neurotoxicity of Hg, even at very low and otherwise safe levels, is further enhanced by co-exposure to Pb, Al, Mn, Cd, Zn, or Fe [99]. In an in vitro study, exposure to a nontoxic level of aluminum hydroxide markedly increased neuronal cell death when combined with Hg [141]. The DJ-1 protein, which is associated with PD and protects cells from stressors, tends to bind Pb and Hg. Exposure to Hg increases the likelihood of developing PD as the genetic adaptation of DJ-1 is affected by Hg toxicity [24]. Metals with a high affinity for sulfhydryl groups, such as Cd, Hg, Zn, and Cu, affect dopamine receptor 2 levels [112]. Metals such as Fe, Al, Cd, Cu, and Mn increase the presynaptic aggregation of α-synuclein, a major histopathological hallmark of PD [99]. Divalent metals, such as Mn and Fe, bind to the C-terminus of α-synuclein with low affinity via an imprecise interface, whereas Cu binds with high affinity to the N-terminus of α-synuclein [142]. It was reported that a mixture of arsenic (As), Cd, and Pb had a synergistic effect on astrocytes in young mice by interfering with the function of the BBB [143]. The same combination of metals (As, Cd, and Pb) induced the accumulation of oxidative stress in neurons through the mobilization of intracellular Ca^2+^, generation of ROS, and triggering extracellular signal-regulated kinase, mitogen-activated protein kinase 3, and c-Jun N-terminal kinases [144].

## 4. Conclusions and Future Perspectives

Metal ions are essential for the proper functioning of various physiological processes in the human body and play important roles in the maintenance of health. Important points in the pathogenesis of metal-related diseases are the equilibrium and composition of these metals in the human body. Numerous studies have elucidated the roles of metals in the pathogenesis of PD and have proposed several possible mechanisms of action. With co-exposure to many neurotoxic metals, the intracellular homeostasis of the metal is disturbed, and intracellular metabolism is altered, resulting in neurodegenerative diseases such as PD. Such changes in the brain lead to the accumulation of α-synuclein and increased oxidative stress, leading to DNA damage and dopaminergic neurodegeneration. A better understanding of the mechanisms of metal toxicity will aid the establishment of novel therapeutics and multidisciplinary approaches to prevent or treat PD.
